# Effects of vitamin D supplementation on apolipoprotein A1 and B100 levels in adults: Systematic review and meta-analysis of controlled clinical trials

**DOI:** 10.34172/jcvtr.2021.21

**Published:** 2021-03-17

**Authors:** Nima Radkhah, Sakineh Shabbidar, Meysam Zarezadeh, Abdolrasoul Safaeiyan, Ali Barzegar

**Affiliations:** ^1^Department of Community Nutrition, School of Nutrition and Food Sciences, Tabriz University of Medical Sciences, Tabriz, Iran; ^2^Department of Community Nutrition, School of Nutritional Sciences and Dietetics, Tehran University of Medical Sciences, Tehran, Iran; ^3^Student Research Committee, Tabriz University of Medical Sciences, Tabriz, Iran; ^4^Nutrition Research Center, Department of Clinical Nutrition, School of Nutrition and Food Sciences, Tabriz University of Medical Sciences, Tabriz, Iran; ^5^Department of Vital Statistics and Epidemiology, Tabriz University of Medical Sciences, Tabriz, Iran

**Keywords:** Vitamin D, Apolipoproteins, Cardiovascular Diseases, Meta-Analysis, Systematic Review, Randomized Controlled Trial

## Abstract

Cardiovascular disease (CVD) is a leading cause of death around the world. According to the studies, apolipoproteins A1 and B100 play crucial role in CVD development and progression. Also, findings have indicated the positive role of vitamin D on these factors. Thus, we conducted the present meta-analysis of randomized controlled trials (RCTs) to demonstrate the impact of vitamin D supplementation on apolipoproteins A1 and B100 levels in adults. PubMed and Scopus databases and Google Scholar were searched up to 21 December 2020. Relevant articles were screened, extracted, and assessed for quality based on the Cochrane collaboration’s risk of bias tool. Data analysis conducted by random-effect model and expressed by standardized mean difference (SMD). The heterogeneity between studies was assessed by I-squared (I2) test. Subgroups and sensitivity Analyses were also conducted. Seven RCTs were identified investigating the impact of vitamin D on Apo A1 levels and six on Apo B100 levels. The findings showed the insignificant effect of vitamin D supplementation on Apo A1 (SMD=0.26 mg/dL; 95% confidence interval (CI), −0.10, 0.61; *P* = 0.155) and Apo B100 (standardized mean difference (SMD)=-0.06 mg/dL; 95% CI, −0.24, 0.12; *P* = 0.530) in adults. There was a significant between-study heterogeneity in Apo A1 (I2=89.3%, *P* < 0.001) and Apo B100 (I2 = 57.1%, *P* = 0.030). However, significant increase in Apo A1 in daily dosage of vitamin D (SMD=0.56 mg/dL; 95% CI, 0.02, 1.11; *P* = 0.044) and ≤12 weeks of supplementation duration (SMD=0.71 mg/dL; 95% CI, 0.08, 1.34; *P* = 0.028) was observed. No significant effects of vitamin D on Apo A1 and Apo B100 levels after subgroup analysis by mean age, gender, study population, dosage and duration of study. Overall, daily vitamin D supplementation and ≤12 weeks of supplementation might have beneficial effects in increasing Apo A1 levels, however, future high-quality trials considering these a primary outcome are required.

## Introduction

Globally, cardiovascular disease (CVD) is a prominent reason of mortality and morbidity.^1^ CVD was has been known to be the western world’s disease, nevertheless, according to the recent evidence, population of low-income countries also suffer from CVDs. based on World Health Organization (WHO) estimation, CVD is the cause of about 17.7 million fatalities, which makes 31% of global deaths in the year 2015.^1^ According to previous reports of Global Burden of Disease (GBD) in 2010 and 2015, CVD was the primary leading cause of mortality and the disability-adjusted life-year (DALY) causing to 46% of all deaths.^2-4^ Atherosclerosis is a low-grade inflammation of the vascular wall caused by lipids deposition and accumulation of macrophages and T-cells resulting from endothelial injury response.^5,6^ It is known that blood circulating lipoproteins particularly low-density lipoprotein (LDL) and high-density lipoprotein (HDL) are key to the pathogenesis of atherosclerosis.^7,8^

Apolipoprotein B100 (Apo B100), as the main protein particle of lipoproteins particularly lipoprotein (a) (Lp (a)), intermediate density lipoprotein (IDL), very low density lipoprotein (VLDL) and LDL, can initiate and progress atherosclerosis.^9-11^ High levels of Apo B containing lipoproteins, chiefly LDL, are associated with the elevated risk of developing atherosclerotic cardiovascular disease (ASCVD).^12^ LDL-cholesterol is one of the key risk factors of ASCVD; thus, it is a preliminary therapeutic goal in both primary and secondary preventions of ASCVD according to global dyslipidemia guidelines.^13-16^ It has been shown that HDL possess anti-inflammatory, antithrombotic, antioxidant and nitric oxide-promoting properties.^17-19^ Also, serum HDL levels show reverse association with the risk of cardiovascular disease and atherosclerosis. Apolipoprotein A1 (Apo A1), the main protein of HDL, is mostly produced in the small intestine and liver.^8^ Excessive cholesterol is transported by APOA1/HDL particles to the liver from peripheral tissues using reverse cholesterol transport pathway.^19^

Vitamin D is a steroid hormone mainly known to be involved in regulating the balance between phosphorus and calcium metabolism by acting on glands, parathyroid kidneys, and intestines.^20^ In the recent years, increasing evidence have been emerged suggesting that that CVD risk is related to the vitamin D deficiency,^21,22^ as well as hypertension,^23^ obesity,^24^ and cancer.^25^ Vitamin D deficiency also accelerates the progression of atherosclerosis.^26^ It has been indicated that most of the populations have lower levels of vitamin D,^27,28^ which could be a potential reversible risk factor for CVD.^29^ Moreover, low HDL-cholesterol, high serum total cholesterol (TC), LDL-cholesterol, Apo B/Apo A1 ratio is associated with low serum 25(OH) D.^30,31^ Moreover, Apo B100/Apo A1 ratio indicates anti-atherogenic /pro-atherogenic lipoproteins balance and could be a reliable risk indicator of cardiovascular conditions when compared with lipid parameters.^32-34^ Various studies have examined the effect of vitamin D on Apo A1 and B100, however, the results of studies are contradictory. Moreover, there was no meta-analysis investigating vitamin D effect on Apo A1 and B100 levels. Therefore, we aimed to perform a meta-analysis to summarize and quantify the effect of vitamin D intake on apolipoprotein A1 and B100 in adults to provide an evidence-based reference for therapeutic approaches.

## Methods

### Protocol and guide

This study was conducted based on the Preferred Reporting Items for Systematic Reviews and Meta-Analysis (PRISMA).^35^

### Search strategy

Investigation criteria were demonstrated using the PICOS (population, interventions, comparisons, outcomes, and study design) which are explained in Table 1. One of the authors (NR) conducted the search in selected databases: PubMed, Scopus and Google Scholar from inception to 21th December 2020. Moreover, the World Health Organization, ClinicalTrials.gov and International Clinical Trials Registry Platform were searched to identify unpublished eligible or ongoing trials. To maximize the comprehensiveness, we studied references of reviews and trials. There were no language limitations in searching databases. Following search pattern was employed to search databases: ((((((((((((1,25 dihydroxyvitamin D3[Title/Abstract]) OR hydroxycholecalciferol[Title/Abstract]) OR vitamin D3[Title/Abstract]) OR hydroxycholecalciferol[Title/Abstract]) OR 25-hydroxyvitamin D2[Title/Abstract]) OR vitamin D2[Title/Abstract]) OR dihydrotachysterol[Title/Abstract]) OR dihydroxycholecalciferol[Title/Abstract])) OR ((“Calcitriol”[Mesh]) OR (“Vitamin D”[Mesh] OR “Ergocalciferols”[Mesh] OR “Cholecalciferol”[Mesh])))) AND (((((((((((((((Randomized controlled trial[Title/Abstract]) OR Randomized control trial[Title/Abstract]) OR Clinical trial Intervention*[Title/Abstract]) OR Randomly*[Title/Abstract]) OR Randomized*[Title/Abstract]) OR Randomised*[Title/Abstract]) OR Control trial[Title/Abstract]) OR “controlled trial”[Title/Abstract]) OR “random”[Title/Abstract]) OR “randomly”[Title/Abstract]) OR “placebo”[Title/Abstract]) OR “assignment”[Title/Abstract])) OR (“Clinical Trial” [Publication Type] OR “Clinical Trials as Topic”[Mesh])) OR ((“Randomized Controlled Trial” [Publication Type]) OR “Randomized Controlled Trials as Topic”[Mesh]))) AND (((((Apolipoprotein*[Title/Abstract]) OR apoprotein*[Title/Abstract]) OR lipoprotein*[Title/Abstract])) OR (((“Apoproteins”[Mesh]) OR “Lipoproteins”[Mesh]) OR “Apolipoproteins”[Mesh])).

**Table 1:** PICOS criteria

Patient/Population: Adults Intervention: Vitamin D supplementation for ⩾ 2 weeks Comparison: Placebo group Outcome: Change in concentration of Apo A1 and B100 in both groups Study design: RCTs

Abbreviations: Apo, apolipoprotein; PICOS, population, interventions, comparisons, outcomes, and study design; RCTs, randomized controlled trials

### Study selection

All RCTs that elaborated on the effect of vitamin D supplementation on Apolipoproteins A1 and B100 were identified. We extracted the abstracts or full texts of all articles, reports, and documents retrieved in advanced search. The inclusion criteria were: RCTs investigating the impact of vitamin D intake on Apolipoproteins A1 and B100 levels.

The exclusion criteria were: uncontrolled trials, trials with treatment duration of < 2 weeks, parenteral administration of vitamin D, co-administration of vitamin D with other interventions, in vitro or animal studies, and series studies or case reports.

### Data extraction

Two reviewers (NR and MZ) performed data extraction, independently. Following information were extracted: first author’s name, location of study, publication year, study population, mean age of participants, sample size, dose and duration of vitamin D supplementation, and the mean ± standard deviation (SD) of Apo A1 and B100 levels in the control and intervention groups at baseline and end of the study. Discrepancies were resolved by the third reviewer (AB).

### Quality assessment

We performed a systematic evaluation of bias using Cochrane criteria which covers the following items: allocation concealment, random sequence generation, blinding of outcome assessors, blinding process, incomplete outcome data, reporting selective outcome, and other potential sources of bias.^36^

### Statistical methods

Data analysis was carried out by STATA 16.0 (Stata Corporation, College Station, TX, US). We used the chi-square test for I^2^ statistics to demonstrate heterogeneity of the mean differences between studies. Significance levels of *P* <  0.10 or I^2^ > 50% were considered to be statistically significant.^37^ We employed random-effect model using restricted maximum likelihood (REML) method to estimate the combined effect size. Meta-analysis was reported as standardized mean difference (SMD) and 95% confidence interval for each variable in Forest plot. Subgroup analyses were carried out to identify the source of heterogeneity between articles in addition to expressing results across various subgroups. Furthermore, sensitivity analysis was performed to demonstrate the effect of exclusion of one single study on the overall results. The presence of publication bias was identified by applying visual inspection of funnel plots, Egger’s and Begg’s tests.^38^ In case of presence of publication bias, trim and fill analysis was performed. *P* of 2-tailed < 0.05 was considered to be statistically significant.

## Results

### Search findings and research flow

**Figure 1:**
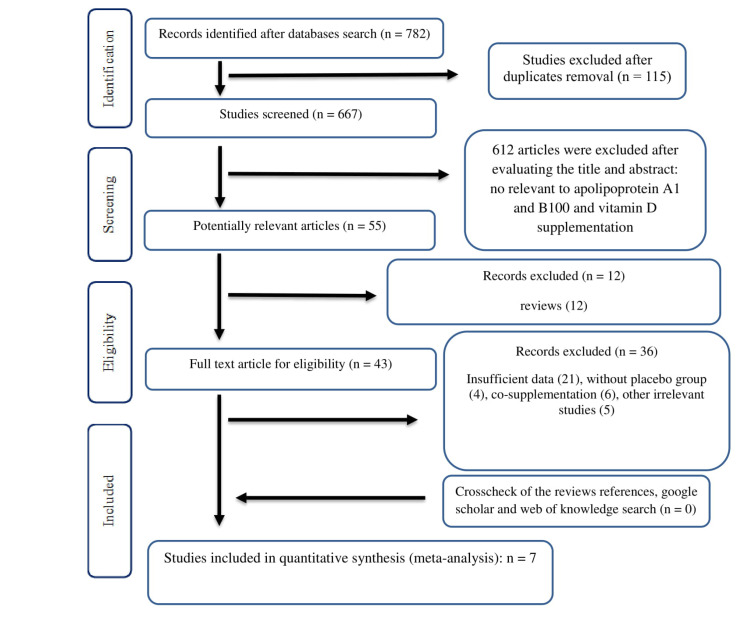
PRISMA flow diagram depicting study selection for systematic review and meta-analysis

A total of 716 studies were found through the primary search. After removing the duplicates, 617 articles went under the evaluation. A number of 571 irrelevant studies were excluded after review of titles and abstracts. The full texts of the 46 remaining studies were evaluated and 36 articles were excluded due to insufficient data (21), lack of placebo group (4), co-supplementation (6), and irrelevant studies (5). Finally, 7 RCTs were included in present meta-analysis. We also cross-checked the references of the relevant articles but no article was found to be included. The PRISMA flow chart of the study is shown on Figure 1.

**Figure 2:**
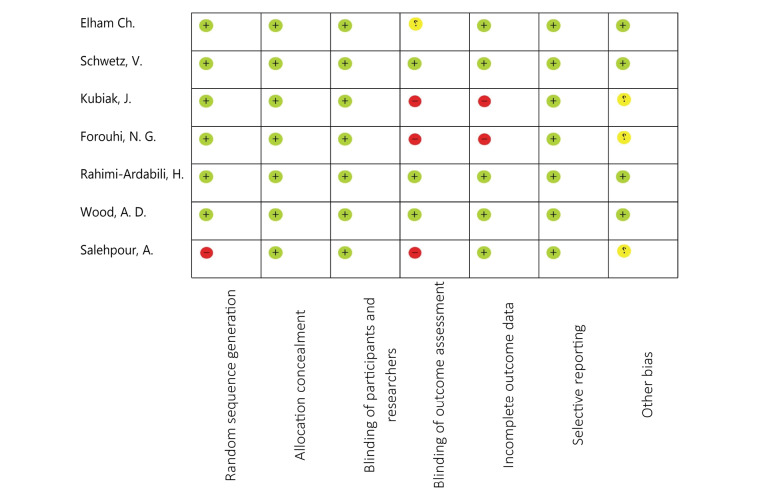
The methodological quality assessment of included studies using Cochrane Collaboration’s Tool

Seven articles reported the impact of vitamin D on Apo A1 level and six on Apo B100 levels. Two trial reported significant increase in Apo A1 and 4 studies have reported significant changes in Apo B100.^39,40^ Study duration varied from 60 to 365 days. The daily supplementation of vitamin D ranged from 400 to 2800 (IU/day) and some was designed with a single dose intervention. Two studies were conducted in UK,^41,42^ three in Iran,^39,40,43^ one in Norway,^20^ and one in Austria.^44^ The detailed characteristics of involved studies are presented in Table 2. The results of quality assessment of the included studies are presented in Figure 2.

**Table 2:** Randomized controlled trials examining the impact of vitamin D supplementation in Apo A1 and B100

**Biomarker**	**Subgroups**	**No. of studies**	**WMD (95% CI)**	***P*** ** value**	**P-heterogeneity**	**I** ^2^ **(%)**
	Total	8	0.26 (-0.10, 0.61)	0.155	< 0.001	89.3
Apo A1 (mg/dL)	Mean age (year)	> 40	5	0.03 (-0.27, 0.34)	0.833	< 0.001	84.3
		≤40	3	0.71 (-0.31, 1.74)	0.174	< 0.001	90.9
	Gender	F	4	0.09 (-0.37, 0.56)	0.689	0.001	82.4
		M/F	4	0.43 (-0.16, 1.03)	0.154	< 0.001	93.7
	Study population	Healthy	5	0.04 (-0.26, 0.33)	0.800	< 0.001	80.9
		Vitamin D deficient	3	0.64 (-0.26, 1.53)	0.163	< 0.001	90.4
	Intervention duration (week)	= 52	2	-0.10 (-0.31, 0.12)	0.375	0.999	0.0
		= 16	2	-0.14 (-0.45, 0.16)	0.359	0.059	72.0
		≤12	4	0.71 (0.08, 1.34)	0.028	< 0.001	86.3
	Vitamin D dosage	Daily	5	0.56 (0.02, 1.11)	0.044	< 0.001	90.7
		Monthly	3	-0.17 (-0.42, 0.08)	0.179	0.126	51.7
	Total		7	-0.06 (-0.24, 0.12)	0.530	0.030	57.1
Apo B100 (mg/dL)	Mean age (year)	> 40	5	-0.09 (-0.30, 0.13)	0.446	0.011	69.5
		≤ 40	2	0.07 (-0.27, 0.40)	0.704	0.664	0.0
	Gender	F	3	-0.04 (-0.23, 0.15)	0.683	0.981	0.0
		M/F	4	-0.06 (-0.38, 0.26)	0.710	0.003	78.3
	Study population	Healthy	5	-0.13 (-0.33, 0.08)	0.217	0.042	59.6
		Vitamin D deficient	2	0.18 (-0.08, 0.44)	0.183	0.898	0.0
	Intervention duration (week)	= 52	2	-0.05 (-0.27, 0.17)	0.650	1.000	0.0
		= 16	2	-0.24 (-0.73, 0.25)	0.335	0.003	88.6
		≤12	3	0.13 (-0.09, 0.36)	0.252	0.789	0.0
	Vitamin D dosage	Daily	5	0.04 (-0.12, 0.19)	0.646	0.776	0.0
		Monthly	2	-0.24 (-0.73, 0.25)	0.335	0.003	88.6

Abbreviations: Apo, apolipoprotein; WMD, weighted mean difference

### Vitamin D supplementation effects on apolipoprotein A1

**Figure 3:**
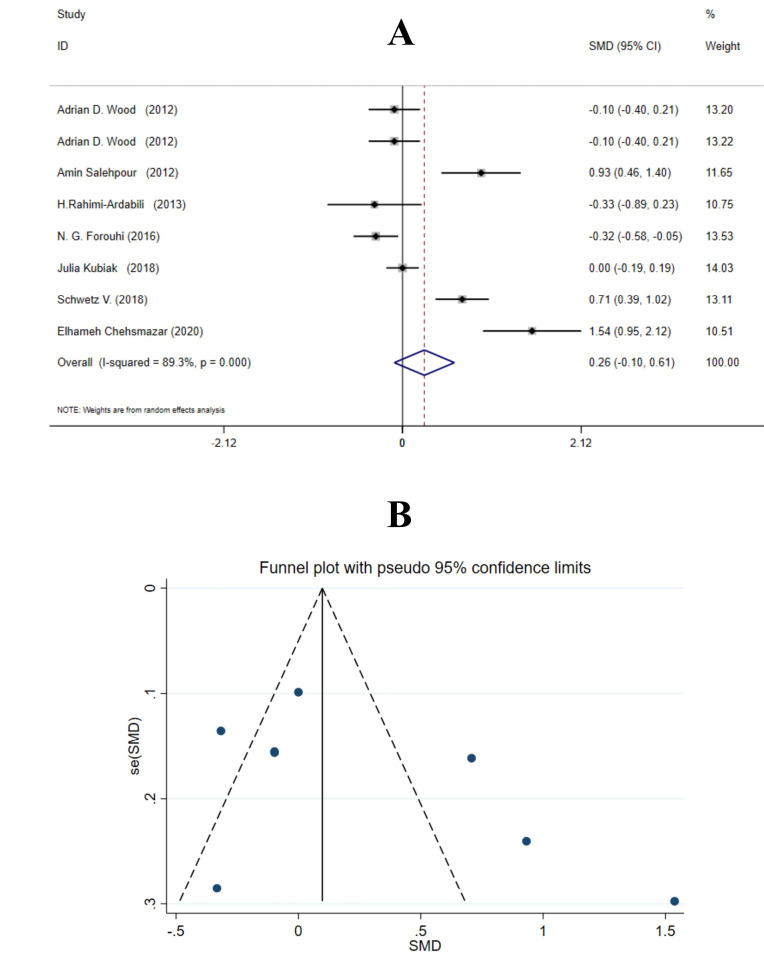
The Forrest plot (A) and Funnel plot (B) of effect of vitamin D on Apo A1 levels

Vitamin D supplementation had no significant effect on Apo A1 levels (SMD = 0.26 mg/dL; 95% CI, −0.10, 0.61; *P* = 0.155) (Figure 3A). Due to the high between-study heterogeneity (I^2^ = 89.3%, *P* <  0.001), intervention duration was identified as source of heterogeneity. Subgroup analyses by suspected variables were conducted including age, gender, study population, supplementation dosage and duration. Daily dosage of vitamin D (SMD = 0.56 mg/dL; 95% CI, 0.02, 1.11; *P* = 0.044) and ≤12 weeks of supplementation (SMD = 0.71 mg/dL; 95% CI, 0.08, 1.34; *P* = 0.028) significantly increased Apo A levels. Findings of the subgroup analyses are presented in Table 3.

**Table 3:** The overall results of subgroup analyses on Apo A1 and Apo B

**Authors (Ref)**	**Publication** **year**	**Sample size** **(control/** **intervention)**	**Country** **(population)**	**Intervention** **(name and daily dose)**	**Duration** **(Day)**	**Presented data**	**Age (control,** **intervention)**	**Results (significant difference)**
Wood A. D.^41^	2012	82/82	United Kingdom (healthy postmenopausal women)	400 IU/d Vitamin D_3_	365	Apo A1 and B100	63.9,63.5	Apo A1: - B100: ↓
Wood A. D.^41^	2012	82/84	United Kingdom (healthy postmenopausal women)	1000 IU/d Vitamin D_3_	365	Apo A1 and B100	63.9,64.1	Apo A1: - B100: ↓
Salehpour A.^39^	2012	38/39	Iran (healthy premenopausal overweight and obese women)	1000 IU/d Vitamin D_3_	90	Apo A1 and B100	37,38	Apo A1: ↑ B100: -
Rahimi-Ardabili H.^43^	2013	26/24	Iran (women with PCOS and vitamin D deficiency)	2500 IU/d = 3*(50.000 IU/20d) Vitamin D_3_	60	Apo A1	27,26.8	Apo A1: -
Forouhi N. G.^42^	2016	111/110	UK (adults who had an elevated risk of type 2 diabetes)	4*(100,000 IU/m) Vitamin D_3_	120	Apo A1 and B100	52.4,52.5	Apo A1: - B100: ↓
Kubiak J.^20^	2018	203/208	Norway (healthy women and men)	100,000 IU loading dose, followed by 20,000 IU/week Vitamin D_3_	120	Apo A1 and B100	51,50	Apo A1: - B100: -
Schwetz V.^44^	2018	84/79	Austria (men and women with arterial hypertension and 25(OH)D < 75 nmol/l)	2800 IU/d Vitamin D_3_	60	Apo A1 and B100	62.1,62.2	Apo A1: - B100: ↑
Elhameh Chehsmazar ^46^	2020	29/30	Iran (obese and overweight men and women with vitamin D deficient under a low-calorie diet program)	2000 IU/d Vitamin D_3_	56	Apo A1 and B100	36.8,38.3	Apo A1: ↑ B100: -

Abbreviations: Apo, apolipoprotein; IU, international unit

No significant publication bias was found according to Egger’s and Begg’s tests (*P* = 0.184 and 0.266, respectively). There was no significant effect of sensitivity analysis on Apo A1. The funnel plot of vitamin D effect on Apo A1 is demonstrated on Figure 3B. Visual assessment of funnel plots indicated no significant asymmetry and publication bias between studies.

### Vitamin D supplementation effects on apolipoprotein B100

**Figure 4:**
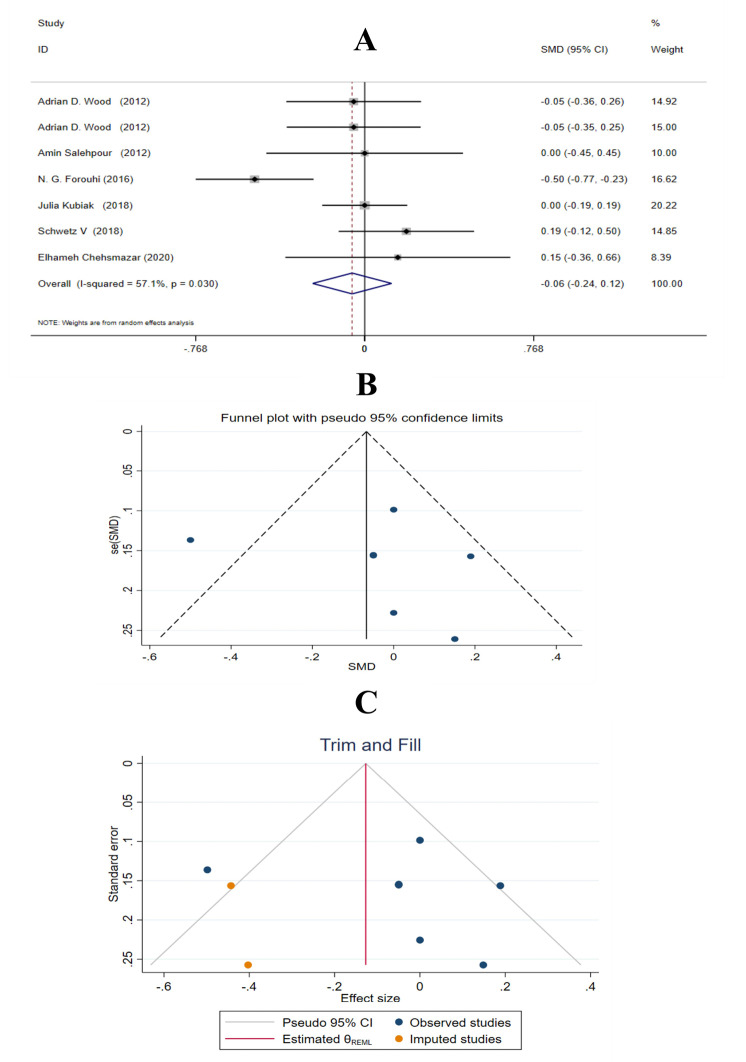
The Forrest plot (A), Funnel plot (B) And Trim and fill (C) of effect of vitamin D on Apo B100 levels

No significant impact of vitamin D supplementation on Apo B100 (SMD = -0.06 mg/dL; 95% CI, −0.24, 0.12; *P* = 0.530) (Figure 4A) was found. There was significant between-study heterogeneity (I^2^ = 57.1%, *P* = 0.030). Age, gender, study population, intervention duration and vitamin D dosage were identified as sources of heterogeneity. There were no significant effects of vitamin D on Apo B100 levels after subgroup analysis (Table 3). In Egger’s and Begg’s tests (*P* = 0.708 and 0.368, respectively) no evidence of small study effect was found. Also, no significant results of sensitivity analysis were observed. The funnel plot of vitamin D effect on Apo B100 showed an asymmetric distribution of studies around the SMD (Figure 4B). Therefore, we performed trim and fill analysis with 2 imputed studies (SMD = -0.127 mg/dL; 95% CI: -0.299, 0.045; *P* > 0.05) (Figure 4C).

## Discussion

Results of present study showed that vitamin D intake had no significant effect on apolipoproteins A1 and B100 levels, however, significant increase of Apo A1 levels in daily dosage of vitamin D and ≤12 weeks of supplementation was observed.

Lipid profile, especially apolipoprotein A1 and B100, are important factors in developing and progressing atherosclerosis and finally CVD.^45^ It has been reported that Apo proteins are as reliable as lipid profile in predicting cardiovascular events.^45^ Several studies have probed the effect of vitamin D Apo proteins. Elham Cheshmazar et al conducted a study with 2000 IU/d of vitamin D supplementation for 8 weeks in vitamin D deficient obese and overweight individuals. Results showed significant increase in Apo A1 levels but not in B100 levels.^46^ Salehpour et al observed that supplementation with 25mcg vitamin D3 improved Apo A1 concentration in overweight and obese women, while it had no significant effect on Apo B100 levels.^39^ Low dosage and low duration of supplementation might be associated with insignificant results on Apo B100 levels. Schwetz et al performed a RCT in middle-aged patients with hypertension and low 25(OH) D levels and the results indicated that high-dose vitamin D supplementation had no effect on Apo A1 levels, however there was an unexpected increase in Apo B levels.^44^ Also, Wood A. D. et al conducted daily supplementation of vitamin D3 (400 or 1000IU) over one year showed no significant effect on APO A1, either.^41^ However the levels of Apo B were significantly improved in both dosage. Lack of insignificant results on APO A1 levels might be due to the lower 25(OH) vitamin D levels in participants as APO A1 levels may respond to an optimum levels of vitamin D. The reason for increasing APO B100 levels in Schwetz et al study is unclear.

Kubiak et al found no significant effect of 4 months supplementation of vitamin D on Apo A1 and Apo B levels.^20^ In another study on individuals at high risk for diabetes, Forouhi et al prescribed four doses of 100,000 IU of vitamin D for 4 months, which significantly reduced Apo B100 levels but showed no significant change in Apo A1 levels.^42^ Kubiak et al and Forouhi et al^20,42^ studies supplemented mega dosages of vitamin D for 120 days. It could be possible that the efficacy of daily supplementation with vitamin D in comparison to mega dosages in more effective in increasing APO A1 levels as the results of present study confirms this hypothesis. According to the studies, the concentration of serum 25(OH) D is potent and independent prognosticator of Apo A1 concentrations.^47-49^ Auwerx et al showed that Apo A1 concentrations positively are associated with 25(OH)D levels.^49^ It has been assumed that in the promoter of the Apo A1 gene, there are vitamin D response elements through which 1,25(OH)2D enhances the transcription of the Apo A1 gene in human hepatoma cells,^50^ however, we observed no significant effect of vitamin D on Apo A1 levels. Moreover, a large-scale RCT in New Zealand on 5110 participants indicated that vitamin D had no significant effect on CVDs.^51^ Based on the results of the subgroup analysis, daily dosage of vitamin D supplementation showed a significant effect on increasing Apo A1 levels. Therefore, daily dose supplementation of vitamin D might be more beneficial than mega dose supplementation. The underlying mechanism for this observation is unknown, however, it is likely that continuous supplementation of this vitamin is effective in genetically regulating of Apo A1 production.^51^ There was also a significant effect of vitamin D supplementation for ≤12 weeks, which is also thought to be due to higher adherence of participants to the recommended protocols in the short-lasting interventions.^41^

The present study is the first systematic review and meta-analysis evaluating the effect of vitamin D supplementation on Apo A1 and B100 levels however it had some limitations. First, the total number of included articles was low which may hypothetically cause unstable estimation of treatment effects. Second, the amount of heterogeneity was notable in studies on Apo A1 and B100, which limits the generalizability of our findings. Third, the study was not registered in registration databases which may introduce source of bias.

## Conclusion

The present review showed that vitamin D supplementation has no favorable effect on Apo A1 and Apo B levels, however, daily supplementation of vitamin D may have beneficial effects on improving Apo A1 levels. Studies with high quality, larger samples size and different health statuses for comprehensive and accurate results are required.
